# A Cross-Sectional Study of Gender Differences in Calorie Labeling Policy among Students: Dietary Habits, Nutritional Knowledge and Awareness

**DOI:** 10.3390/nu15040879

**Published:** 2023-02-09

**Authors:** Naif M. Alotaibi, Ghedeir M. Alshammari, Kholoud B. Alabdulkarem, Abdulaziz A. Alotaibi, Mohammed A. Mohammed, Athoug Alotaibi, Mohammed Abdo Yahya

**Affiliations:** 1 Department of Food Science and Nutrition, College of Food and Agricultural Science, King Saud University, P.O. Box 2460, Riyadh 11451, Saudi Arabia; 2 Department of Social Studies, College of Arts, King Saud University, Riyadh 12372, Saudi Arabia; 3 Public Administration Department, College of Business Administration, King Saud University, Riyadh 11543, Saudi Arabia

**Keywords:** nutrition, public health, calorie labeling, food habits, public policy

## Abstract

Calorie labels may be the most important predictor of dietary choices among college students. The Saudi Food and Drug Authority (SFDA) has imposed calorie labels on the menus of restaurants and cafes. The current study looked at how the calorie labeling policy affects Saudi male and female students’ dietary habits, nutritional knowledge, and awareness. The study included 802 students (360 males and 442 females) from Saudi Arabia’s King Saud University, ranging between 18 and 35 years. Between December 2020 and October 2021, a cross-sectional, electronic, approved and validated survey was conducted to collect data on gender socio-demographic variables, food habits, and nutritional knowledge and awareness, in accordance with the food policy stated. The collected data were analyzed using descriptive statistical analysis. The Likert scale was used to determine the level of awareness and the food habit scores, and the Mann-Whitney U-test was used to determine the differences between the males and females. Spearman’s correlation coefficient and simple regression analysis were performed to determine the association between the demographic factors and nutritional knowledge and the awareness of males and females. The results demonstrated that, with the exception of living situations, males and females differed significantly (*p* ≤ 0.01) in their socio-demographic characteristics. When asked about their food habits after the implementation of calorie labeling, the majority of respondents (>50%) gave negative responses, with a significant difference observed between maintaining body weight (*p* ≤ 0.05) and gaining weight (*p* ≤ 0.01). According to the Likert scale, there was a significant difference between males and females in terms of knowledge (*p* ≤ 0.01) and awareness (*p* ≤ 0.05). An average of 80.53% of males had very high knowledge (4.07) and 65.65% had medium level (3.24) awareness of calorie labeling, while 83.73% of females had very high knowledge (4.17) and 66.50% had medium level (3.32) awareness of calorie labeling. The socio-demographic and lifestyle variables were significantly and positively or negatively associated with calorie label utilization and varied between respondents, according to the Spearman correlation coefficients (*r*) and simple linear regression analysis. The number of factors that negatively impacted the males’ knowledge and awareness was greater than that of the females. In conclusion, among college students, there were numerous gender differences in the demographic and social characteristics. The respondents’ knowledge was insufficient, with females outperforming males.

## 1. Introduction

An individual may be predisposed to obesity by lifestyle, genetics, environment, or a mix of these factors [1]. Energy expenditure is dropping, and the lifestyle is becoming more sedentary in Saudi Arabia, particularly in the cities, where many families commute by vehicle and their children spend a lot of time watching television, using computers, and playing video games [1]. Therefore, Saudis’ physical activity has been significantly impacted by changes in lifestyle and socioeconomic level [2]. Researchers discovered a sharp decline in Saudis’ physical activity patterns over the past few decades [3]. Only 25% of Saudi individuals were deemed to be physically active, and 40% were considered inactive, according to a survey conducted on Saudi adults [4]. There are gender inequalities in physical activity among Saudis [4], which may be a result of the country’s distinct culture. Sedentary behavior increases the risk of type 2 diabetes [5], obesity, and cardiovascular disease [6]. According to a study by Al-Nuaim et al. [7], lifestyle factors have an impact on the prevalence of obesity among students in Saudi Arabia’s eastern province. According to Al-Hazzaa et al. [8], adolescents in Saudi Arabia who have irregular sleep patterns are more likely to be obese. Watching TV is linked to the frequency of obesity among schoolchildren in the Riyadh region, according to a different study by Al-Ghamdi [9]. According to Pengpid and Peltzer [10], sociodemographic characteristics, including gender, age, and socioeconomic level, as well as social factors such as a lack of social support, wealth, and eating habits are linked to overweight and obesity in university students and adults.

Since the 1970s, the Gulf region’s cultural, social, and economic changes have been linked to obesity. One probable explanation for the dramatic rise in obesity rates is a shift toward a more Western-style diet [2]. Furthermore, the Saudi population has recently been observed to have been increasing their consumption of meats and animal products, resulting in a scarcity of fruits and vegetables [11]. Obesity is one of the most serious problems related to poor eating habits and food consumption [12,13]. Obesity has become a major health problem in the last two decades, connected with a lack of physical activity and unhealthy eating habits [14]. Obesity is linked to various lifestyle problems, including hypertension, heart ailments, and diabetes [11]. Furthermore, they discovered that 75% of Saudi women were overweight, and 44% were obese [11]. However, in response to this, government organizations can develop a wide range of projects and strategies. One technique includes providing nutrition and calorie information on restaurant and fast-food menus [15].

Transitioning from high school to college can be difficult for young adults due to the fact that it is determined by establishing routines, habits, and preferences that may persist throughout adulthood [16]. According to a cross-sectional study, adulthood relates to decreased fruit and vegetable consumption, increased fast food and soft drinks consumption, and decreased physical activity levels [16]. Moreover, many young people gain weight during college, particularly during their first year [17]. On 1 January 2019, the Saudi Food and Drug Authority (SFDA) mandated calorie labeling on restaurant and café menus. The goal of such an endeavor is to encourage informed public decision-making regarding healthy food options, as well as to improve overall health predictably.

The primary goal of calorie labeling is to assist consumers in making smarter dietary choices, which often include fewer calories and contribute to a healthier lifestyle. Furthermore, calorie labeling may improve transparency and accountability in restaurants regarding the food they serve [18]. Bollinger et al. [19] analyzed the impact of calorie labeling and concluded that the nutritional labeling of information on food goods enhances consumer health and correlates to a moderate reduction in calorie purchases. Christoph et al. [20] performed research on the effects of calorie labeling in university dining halls and discovered that including calorie information in university dining hall menus resulted in a significant reduction in students’ consumption of high-calorie meals. However, as the calorie labels were removed, the higher calorie value of the purchased meals gradually grew.

According to Driskell et al. [21], 88% of college students are more likely to read published calorie information and are more likely to adjust their dietary decisions after reading calorie labels. According to Bates et al. [22], females were more inclined to choose a meal based on the published calorie content; in other words, displaying calorie content does not substantially affect the types of meals that men order. Similarly, research showed that females are more likely than males to study calorie labels and nutrition information [23]. In order to address obesity and poor diet, accurate and understandable nutrition labeling should be prioritized as one of many effective public health strategies which will enable consumers to make educated dietary decisions [24]. Before initiating this study, the authors anticipated that the calorie labeling mandated by Saudi authorities would positively impact many people’s nutritional status. In the current study, a cross-sectional survey was conducted to investigate the gender differences in the calorie labeling policy on food habits, knowledge, and awareness, as well as the determinant factors associated with knowledge and awareness.

## 2. Materials and Methods

### 2.1. Study Sample and Calorie Label

Between November 2019 and February 2020, 802 students (360 males and 442 females) from King Saud University in the Kingdom of Saudi Arabia were engaged in a cross-sectional study. Those who expressed interest were informed regarding the study and also provided informed consent at enrollment, following the guidelines detailed in the Helsinki Declaration. However, based on the selection criteria, there were only 802 candidates that were able to participate in the study safely. Those who followed any form of a prescribed diet, including diet treatment, as well as those suffering from any chronic disease (hypertension, diabetes, heart disease, renal disease, high cholesterol, or liver disease), were excluded from the selection procedure. The response rate among the participants was 100%. The calorie label and information for each item were placed on the restaurant and café menus.

### 2.2. Methods

An expert committee at King Saud University’s Department of Food Science and Nutrition, Faculty of Food Science and Agriculture, validated and approved a structured questionnaire. In addition, the Institutional Review Board (IRB) at King Saud University reviewed the research questionnaire. The questionnaire was pretested and explained to all of the respondents to ensure clarity and ease of understanding. To collect the data, three sections were used:

1. Data on demographic characteristics: gender, marital status, age, kind of college, educational level, total monthly income, and housing circumstances.
2. The consequences of calorie labeling on food habits. In respect of this, the list of questions was included.
3. The consequences of calorie labeling on respondents’ level of knowledge and awareness were included.

#### 2.2.1. Food Habits Survey Attributes

A questionnaire was used to assess the respondents’ opinions regarding food habits, after the implementation of calorie labeling, with the answer “Yes” or “No”. Then, the collected data were analyzed using Chi-Square to determine the differences between the males and females. The questions include: when ordering from restaurants, do you select the type of food based on the fact that it has less calories than other types of food? Do you use the calorie information on menus in order to maintain your body weight? Do you use the calorie information on menus in order to lose weight? Do you use the calorie information on menus in order to gain weight? Do you use the calorie information on menus in order to reach your daily calorie intake requirements? Do you use the calorie information on menus due to medical reasons? Do you count your daily calorie intake?

#### 2.2.2. Knowledge Survey Attributes

A 5-point Likert scale was used to assess the respondents’ opinions regarding calorie label knowledge, with 1 representing “strongly disagree” and 5 representing “strongly agree.” The respondents were asked a series of questions, which related to the following topics:

Calories are a unit of measurement for the amount of energy in food; keeping track of the number of calories taken in food can help you achieve your optimum weight; calories are found in vitamins and minerals; calories are found in carbohydrates, proteins, and lipids; in terms of calories, provided fat and carbohydrates vary; the daily calorie requirements of an individual are affected by various factors, including weight, height, age, gender, and physical activity; the Saudi Food and Drug Authority ordered restaurants and coffee shops to include calorie information on their menus when ordering from a restaurant; it is a good idea to check the calorie information on the menu; calorie labels on restaurant menus are simple to understand; adults require approximately 2000 calories per day on average; individuals’ daily calorie requirements vary from one another; knowing the number of calories consumed aids in balancing added and utilized energy in the body. Thereafter, the mean score of each question was collected and a Whitney U-test was used to determine the differences between the males and females.

#### 2.2.3. Awareness Survey Attributes

A 5-point Likert scale was used to assess the respondents’ opinions regarding the impact of calorie labels on their awareness, with 1 representing “strongly disagree” and 5 representing “strongly agree.” The respondents were asked a series of questions, which related to the following topics:

When it comes to food, I am unconcerned with eating healthy; I am very concerned that the food I eat is nutritious; I eat food that I enjoy regardless of whether it is healthy or not and the meals I eat must be low in fat; I always eat a well-balanced and healthy diet; my meal must be rich in vitamins and minerals; I do not care whether the snacks are nutritious or not; I do not avoid foods that raise my cholesterol; I do not want to think about whether the food I am eating is beneficial for me or not; I am willing to forego certain items in order to focus on a healthy diet; it is critical to understand how to eat a healthy diet; other individuals I feel are more concerned with good eating than I am; I believe that eating fruits and vegetables five times per day is essential; I am concerned about not ingesting too much food; I am concerned about eating a well-balanced diet; I am quite concerned about eating my meals regularly; and I am concerned about not in taking excessive amounts of sugar. Thereafter, the mean score of each question was collected and a Whitney U-test was used to determine the differences between the males and females.

#### 2.2.4. Ethical Standard Disclosure

The Institutional Review Board (IRB) at King Saud University reviewed the research proposal. As a result, the researchers received approval for this study from the IRB with the reference number: KSU-HE-19-172.

#### 2.2.5. Statistical Analysis

The statistical package for the Social Sciences-SPSS-version 20 was used for the purposes of the statistical analysis (Somers, NY, USA). Descriptive statistics for the respondents’ socio-demographic features and food habits were provided as frequencies. Thereafter, Chi-square was used to determine the gender differences. Knowledge and awareness, according to the Likert scale, were determined as means (SD) and standard deviations. Thereafter, a Mann–Whitney U-test was used to detect the differences in the males’ and females’ knowledge and awareness scores. The Spearman correlation coefficient was utilized in order to determine the relationships between the demographic factors and the respondents’ knowledge and awareness. Furthermore, a simple regression analysis was performed in order to establish the relative impact of the independent variables on the changing levels of the dependent variables.

## 3. Results

### 3.1. Respondents Frequency Distribution (n = 802) by Demographic and Social Factors

Table 1 shows the frequency distribution of the respondents according to the demographic and social characteristics. A total of 802 students (360 males and 442 females) completed the questionnaire. According to the data, significant (*p* ≤ 0.05 and *p* ≤ 0.01) variations were observed in the demographic and social characteristics between the males and females. More than half of the respondents were female (55.1%) compared to males (44.9%). Based on marital status, only 7.8% of the males were married compared to 20.8% of females. The majority of both males and females were aged <25 years; in addition, many of them were studying in science colleges, followed by arts and health colleges. The majority of the respondents possessed bachelor’s degrees, with a monthly income of <2000 SR, and lived with their families. However, a small percentage of the participants did live in university housing.

### 3.2. The Effects of Calorie Labeling on Food Habits, Knowledge and Awareness, among Male (n = 360) and Female (n = 442) Respondents

The effect of calorie labeling on food habits, as shown in Table 2, revealed that slightly more than half of the males (55.3%) and females (52.3%) described the calorie labeling as not being a useful tool to determine food habits based on the calorie level. Moreover, a significantly (*p* ≤ 0.05) higher percentage (58.3%) of males disagree with the fact that the calorie labeling menu is not a useful tool for maintaining body weight than females. On the other hand, a significantly (*p* ≤ 0.05) higher percentage (92.1%) of females disagree with the fact that the calorie labeling menu is not a useful tool for gaining weight than males. Furthermore, despite the higher percentage of females, there was no significant difference between males (56.4%) and females (60.4%) who did not use calorie information on menus to meet their daily calorie requirements. The majority of the respondents (93.9% of males and 94.6% of females) advised that they did not use calorie information on menus for medical reasons. In comparison, 68.1% of males and 71.0% of females did not track their daily calorie intake, but there was no statistically significant difference.

According to the Likert scale, Table 3 shows the influence of calorie labeling on the respondents’ knowledge and awareness levels. Females, on average, made more effort than males to gain knowledge (*Z* = −3.912, *p* = 0.004) and awareness (*Z* = −2.322, *p* = 0.013) about the calorie labeling policy. More than half of the males and females had inadequate knowledge that vitamins and minerals have calories. Moreover, when compared to females, 68.34% of males had inadequate knowledge of whether the daily calorie requirements of an individual are affected by weight, height, age, gender, and physical activity. According to the Likert scale, other knowledge attributes of the males and females’ scales were either very high or high with respect to a significant difference between genders in certain cases.

Regarding the effect of calorie labeling on the respondents’ awareness, according to the Likert scale, the awareness attributes scales were low. Both sexes were unaware that they used to eat food that they enjoyed regardless of whether it is healthy or not (*Z* = −2.967, *p* = 0.003); whether they always ate a well-balanced and healthful diet (*Z* = −1.91, *p* = 0.056); whether they do or do not care that the snacks are nutritious or not (*Z* = −2.249, *p* = 0.025); and whether they believe that other people are more concerned with good eating than them (*Z* = −1.179, *p* = 0.238). Certain scores showed a significant difference and were classified as strongly agreed, agreed, or neutral regarding other awareness traits.

### 3.3. Determinant Factors Associated with Male (n = 360) and Female (n = 442) Awareness and Food Habits

According to the Spearman’s correlation coefficient and simple regression analysis between the respondents’ knowledge attributes as dependent variables and socio-demographic characteristics as independent variables, Table 4 and Table 5 show the factors influencing male and female knowledge attributes on calorie labels. As an independent variable, the males’ marital status (Table 4) was inversely associated with the knowledge that the participants’ daily calorie requirements are affected by various factors, including weight, height, age, gender, and physical activity. Moreover, with age, the males had inadequate knowledge that the SFDA ordered restaurants and coffee shops to include calorie information on their menus. The males’ living status was significantly and inversely associated (*p* ≤ 0.01) with many of the knowledge attributes. However, marital status and monthly income entailed an adverse and significant influence (*p* ≤ 0.01 or 0.05) on the females’ (Table 5) knowledge that calories are a unit of measurement for the amount of energy in food that is independent of the educational level—which, in turn, possessed an adverse and significant influence (*p* ≤ 0.01) on the fact that calories are found in vitamins and minerals. Furthermore, the females’ marital status also contributed to rendering them unaware that in terms of calories provided, fat and carbohydrates vary.

Table 6 and Table 7 shows the factors influencing male and female awareness traits on calorie labels, respectively. In respect to the males (Table 6), all of the independent factors except living conditions resulted in a negative effect (*p* ≤ 0.01 or 0.05) on their awareness to make healthy food or to follow a balanced and healthy diet. However, for females (Table 7), all of the independent factors, except monthly income and living status, had a negative impact (*p* ≤ 0.01) on eating nutritious foods, where age, specifically, engendered a negative effect (*p* ≤ 0.01) on the intake of a balanced and healthy diet. The males’ awareness that foods low in fat or high in vitamins and minerals were adversely affected (*p* ≤ 0.01 or 0.05) by marital status, age, and educational level. In addition, such variables also negatively and significantly (*p* ≤ 0.05) affected the females’ intake of high vitamins and minerals. The females’ living status adversely (*p* ≤ 0.05) affected their awareness of caring whether the snacks were nutritious, avoiding meals that boost their cholesterol, or considering whether the food they ate was beneficial to them. Marital status, age, and monthly income negatively correlated (*p* ≤ 0.01) with the males’ willingness to forsake specific products in order to focus on a healthy diet. The females’ age, educational level, and monthly income adversely (*p* ≤ 0.01 or 0.05) influenced their awareness regarding eating a well-balanced diet, eating meals regularly, and not drinking excessive amounts of sugar. However, regarding the males’ marital status, age, and educational level, it was found that these factors negatively (*p* ≤ 0.01 or 0.05) influenced their awareness of eating meals regularly.

## 4. Discussion

Adolescent health-promoting lifestyles are gaining popularity around the world [25]. For example, studies conducted in the United States (US) and in European countries examined university students’ health-promoting activities, particularly their physical activity and eating habits or diet [26]. Individual food choices boost overall health; therefore, providing a supportive environment on a national scale is critical in order to encourage and expand healthy consumer preferences and habits [27]. National policies and regulations on nutrition and food production may help to improve dietary habits at the population level in Saudi Arabia [27]. Thus, it is worthwhile to investigate the consequences of the SFDA’s implementation of Vision 2030, which mandated all restaurants and cafes to show the calories for their menu items in order to reduce the problems associated with poor eating habits [28]. In the current study, a cross-sectional survey was conducted to investigate the influence of the calorie label policy on adult Saudi males and females’ knowledge, awareness, and food habits. In this study, a cross-sectional survey was conducted to investigate the gender differences in the calorie labeling policy on food habits, knowledge and awareness, as well as the determinant factors associated with knowledge and awareness.

The current study showed that the frequency distribution of males (*n* = 360) and females (*n* = 442), according to the demographic and social variables, revealed that the respondents significantly differed in all characteristics, as indicated by the Chi-square test. Furthermore, the results showed that the majority of both sexes were either single, young (<25 years), had a bachelor’s degree, or low income. These variations in the demographic and social characteristics could be attributed to differences in living environments, as well as the differences in tribe [29].

According to the Chi-square analysis, the food habit attribute differed between the sexes, with the habit of using calorie labels to maintain or gain weight significantly differing between males and females, and both disagree with this. According to Orbell and Verplanken [30], the repetition of behavior in a specific context leads to the formation of a behavioral habit. In the current study, the majority of the college students were concerned about eating a well-balanced diet, with a larger percentage of women concerned about not taking excessive amounts of sugar. The habit of choosing fast food was repeatedly linked with the influence of previous consumption habits, as reported by Anderson and Mirosa [31]. Our findings indicate a strong relationship between attitude and behavior, as well as the use of calorie information on restaurant menus. This finding is supported by Kim et al. [32], who stated that attitude determines consumer intention. In addition, Dunn et al. [33] demonstrated that intention influences attitude in situations where fast food is perceived to provide convenience and satisfaction.

Food calorie knowledge is useful for achieving better food selection and healthy weight loss, as well as for aiding with long-term consequences in respect to overall health [34]. According to the Likert-scale, the effect of calorie labeling on meal ordering behavior (knowledge) revealed that, with some exceptions, both males and females’ knowledge of calorie labeling is acceptable. The inadequate level of knowledge could be due to the fact that most respondents were undergraduate students. In addition, more specifically, more than 20% were from the College of Arts and Humanities. In this study, the results regarding this type of inadequate knowledge are in agreement with the results of Ashoori et al. [35], who stated that students who majored in the natural sciences possessed a much better chance of attaining a higher knowledge score than those who majored in literature and the humanities. This could be due to the fact that the natural sciences courses were more likely to cover food- and nutrition-related themes than other majors [35].

According to this study, the females were more likely to limit their calorie intake than their male counterparts. This disparity in knowledge between males and females appears to be caused by males with limited knowledge of the average daily calorie requirement. Roseman et al. [36] discovered that providing calories on menus positively affected the investigated participants. Further, they reported that females were found to process nutrition label information better than males and possess higher nutritional knowledge scores [36]. Indeed, this may be due to the fact that they are more conscious of calories when planning meals for the family. Other possible attributing factors for the observed gender disparities could be that females skipped meals more frequently than males and had greater concerns about body weight and image, whereas males, by eating out with peers more frequently, consumed more fast-food-type food [36]. Males are often more active than females, which influences the amount of food consumed and the food choices made [37]. In line with a prior study by Gerend [23], the current investigation discovered that changes in calories were more noticeable in females than in males. Moreover, Hendrie et al. [38] found that females had greater nutritional knowledge scores than males. In contrast, Affram and Darkwa [39] reported that the knowledge, interpretation, and application of food label information did not differ significantly by gender, but did by age.

The results regarding the effect of calorie labeling in respect to the awareness level of males (*n* = 360) and females (*n* = 442), according to the Likert scale, the awareness of the calorie label scale was significantly higher for females than males. Despite the average medium level of awareness among participants, according to Alkhaldy et al. [40], even though Saudi consumers expressed strong support and awareness for the policy requiring restaurants to provide calorie information on menus, the same respondents expressed a lower likelihood of eating at a restaurant that provided such information on menus. In agreement with the current findings, Campos et al. [41] reported that females understand nutrition label information better than males, possibly because they are more concerned regarding calories when preparing family meals. Moreover, Satia et al. [42] reported that females’ food choices were more influenced by food label use than males. Furthermore, they trusted and utilized food label information to a greater degree. According to the current findings, the Saudi-Arabian-specific factors associated with food labels differ between male and female adults, implying that females review label ingredients more frequently and carefully than men. Additionally, the results indicated that females, when buying food, are more likely to rely on nutrition fact labels, health claims, ingredient lists, and portion sizes than men. According to the study of Campos et al. [41], females check the bulk of the label’s ingredients more frequently than males.

The Spearman’s correlation coefficient and simple regression analysis was used to determine the factors associated with the respondents’ knowledge and awareness attributes as dependent variables and the socio-demographic characteristics as independent variables. According to the data obtained in this study, the main determinants associated with the traits of knowledge regarding calorie labels of the respondents were marital status, age, and living status for males, while for females, marital status, age, and monthly income were more influential. However, the males’ living status did not affect their food habit traits following the utilization of calorie labels due to the fact that other variables adversely affected their attributes instead. In respect to the females, all of the socio-demographic factors were inversely associated with the awareness attributes following the introduction of calorie labels.

In contrast to the present finding, Greene et al. [43] reported that high-income participants were nearly three times more likely to use calorie information in order to aid with purchasing decisions concerning the use of calorie menu labels. In addition to income, age and education were also associated with calorie menu labels. Moreover, it was observed that marital status was significantly associated with nutrition labels [44]. In agreement with the present results, Neumark-Sztainer et al. [45] reported that regular family meals might create healthy eating habits and act as role models for healthy food choices. Family structure and socioeconomic status have also influenced nutritional status [46].

In comparison to older people, the majority of the respondents were young, and they were expected to be aware of calorie labeling. This finding is consistent with Petrovici and Ritson [47], who discovered that older age groups were less likely to read nutrition labels. Although older people may be more interested in healthier eating than younger people, they are more likely to possess a visual impairment and may find it difficult to read nutrition labels with small fonts [48]. Moreover, education and monthly income were associated with label awareness and food behavior features. Those with a higher level of education were more likely to utilize food labels. This finding supports the previous findings of Jacobs et al. [49] and Grunert et al. [50], who reported that individuals with a higher education level might have better knowledge and a better grasp of health-related information than those with a lower education level. Internal influences, such as education, are critical for the purposes of reading, comprehending, and using food label information. Further, Macanda [51] reported that less literate and less educated consumers are vulnerable and frequently illogical regarding food and health issues; therefore, they do not profit from the majority of the product information on a food label.

College students were more likely than high school students to use food labels [52], indicating that consumers with a higher level of education were more likely to use food label information [53]. With a few exceptions, income has been found to favor respondents’ awareness of food labels and purchasing decisions. This finding supports previous findings that higher-income people are more likely than lower-income people to read food labels [44,54]. According to the current study, marital status was also significantly related to food labels, whether in terms of knowledge or awareness, which is consistent with Graham and Jeffery [44]. This was supported by Hanson and Chen’s [35] finding, who reported that adolescent nutritional status was proven to be influenced by family structure and socioeconomic level. Barreiro-Hurlé et al. [55] described the geographical influence on nutritional behaviors and dietary choices in the United States, and they discovered that nutritional behaviors differed depending on the residing regions.

## 5. Conclusions

The respondents’ demographic and social characteristics differed significantly. Gender differences in the effects of calorie labels on food habits were found, with the females outperforming the males. With a few exceptions, both genders’ Likert scale food knowledge and awareness scores were reasonable, with females making more efforts to have knowledge and to be aware than males. Among Saudi male and female adults, socio-demographic and lifestyle characteristics were substantially associated with nutrition label use. With the exception of educational level and monthly income, the socio-demographic characteristics were negatively associated with males’ calorie label knowledge and awareness. Moreover, with the exception of living status, females’ socio-demographic characteristics were significantly and negatively associated with food label knowledge and other factors related to their awareness. The results of the study offer critical insights into the variables impacting the use of food labels knowledge and awareness among a nationally representative sample of adult males and females. These factors must be taken into account by public health professionals, food producers, and policymakers when creating, organizing, and executing an integrated strategy for effective and culturally appropriate nutrition education and food labeling. In addition, for education campaigns, the adoption of a uniform format with streamlined information, a readable typeface, and clear language and symbols could improve Saudi consumers’ use of food and nutrition labels. Moreover, males and females have different factors influencing how they use food labels; therefore, it could be advantageous to explore targeting them separately.

### Limitations

This study was only open to current students at King Saud University. The sample size reflects the number of people who agreed to take the survey. Furthermore, all of the participants who possessed chronic diseases or ate special meals for a specific reason were excluded. Another possible explanation is that individuals would have participated if further incentives were added. Anthropometric measures and physical activity data were not gathered. Lastly, there was also a lack of ability to detect causal relationships or draw broad generalizations due to the cross-sectional study design.

## Figures and Tables

**Table 1:** Frequent distribution of Male (*n* = 360) and Female (*n* = 442) respondents by demographic and social characteristics.

	Male		Female		Chi-Square	*p*-Value
Characteristics	Frequency	Percentage	Frequency	Percentage		
Marital Status						
Single	332	92.2	350	79.2	26.501 **	0.0006
Married	28	7.8	92	20.8		
Age						
<25	305	84.7	284	64.3	45.599 **	0.0001
25–30	28	7.8	104	23.5		
>30	27	7.5	54	12.2		
College						
Health	76	21.1	128	29.0	26.234 **	0.0001
Science	202	56.1	168	38.0		
Art and Humanity	82	22.8	146	33.0		
Educational level						
Bachelor	317	88.1	288	65.2	56.136 **	0.003
Postgraduate	43	11.9	154	34.8		
Total monthly income						
<2000 SR	233	64.7	271	61.3	22.557 **	0.0001
2000–4999 SR	80	22.2	61	13.8		
≥5000 SR	47	13.1	110	24.9		
Living status						
Living with Family	314	87.2	404	91.4	3.698	0.054
Living away from Family	46	12.8	38	8.6		

** *p* ≤ 0.01.

**Table 2:** Effects of calorie labeling on food habits of Male (*n* = 360) and Female (*n* = 442) respondents.

Food Habits		Male		Female		Chi-Square	*p*-Value
		Yes	No	Yes	No		
When ordering from restaurants, do you select the type of food based on the fact that it has less calories than other types of food?	F	161	199	211	231	0.725	0.394
	%	44.7	55.3	47.7	52.3		
Do you use the calorie information on menus in order to maintain your body weight?	F	150	210	216	226	4.148 *	0.042
	%	41.7	58.3	48.9	51.1		
Do you use the calorie information on menus in order to lose weight?	F	170	190	216	226	0.215	0.643
	%	47.2	52.8	48.9	51.1		
Do you use the calorie information on menus in order to gain weight?	F	63	297	35	407	16.981 **	0.0004
	%	17.5	82.5	7.9	92.1		
Do you use the calorie information on menus in order to reach your daily calorie intake requirements?	F	157	203	175	267	1.321	0.250
	%	43.6	56.4	39.6	60.4		
Do you use the calorie information on menus due to a medical reason?	F	22	338	24	418	0.170	0.680
	%	6.1	93.9	5.4	94.6		
Do you count your daily calorie intake?	F	115	245	128	314	0.837	0.360
	%	31.9	68.1	29.0	71.0		

F, frequency, * *p* ≤ 0.05 and ** *p* ≤ 0.01.

**Table 3:** The effect of calorie labeling on knowledge and awareness of Male (*n* = 360) and Female (*n* = 442) respondents according to the Likert scale.

Statement	Males			Females			*Z*	*p*-Value
	Mean Score (SD)	Percent	Awareness/Food Habit Level	Mean Score (SD)	Percent	Awareness/Food Habit Level		
Knowledge								
Calories are a unit of measurement for the amount of energy in food	4.01 (0.87)	85.00	Very High	4.45 (74.31)	89.02	Very high	−3.015	0.003 **
Tracking of the number of calories taken in food can help you achieve your optimum weight	3.92 (1.00)	72.70	High	4.22 (0.70)	85.10	Very high	−0.074	0.941
Calories are found in vitamins and minerals	2.77 (1.34)	56.80	Low	2.21 (1.46)	50.60	Low	−3.099	0.002 **
Calories are found in carbohydrates, proteins, and lipids	4.61 (0.59)	90.60	Very High	4.37 (0.74)	87.40	Very high	−1.237	0.216
In terms of calories provided, fat and carbohydrates vary	3.78 (1.47)	70.70	High	3.56 (1.12)	66.09	Medium	−1.652	0.099
The daily calorie requirements of an individual are affected by various factors, including weight, height, age, gender, and physical activity	3.50 (1.09)	68.34	Medium	4.51 (0.86)	91.80	Very high	−0.547	0.585
The Saudi Food and Drug Authority ordered restaurants and coffee shops to include calorie information on their menus	4.37 (0.68)	85.04	Very High	4.80 (0.59)	96.90	Very high	−1.598	0.110
When ordering from a restaurant, it is a good idea to check the calorie information on the menu	4.29 (1.01)	86.30	Very High	4.11 (0.95)	82.90	Very high	−0.702	0.482
Calorie labels on restaurant menus are simple to understand	4.27 (0.71)	90.20	Very High	4.50 (0.75)	87.10	Very high	−1.510	0.131
Adults require approximately 2000 calories per day on average	4.18 (0.81)	81.00	Very High	4.34 (0.71)	89.90	Very high	−4.502	0.0001 **
Individuals’ daily calorie requirements vary from one another	4.79 (0.57)	96.80	Very High	4.71 (0.66)	93.40	Very high	−0.062	0.950
Knowing the number of calories consumed aids in adding balance and utilized energy in the body	4.31 (0.77)	82.90	Very High	4.25 (0.83)	84.60	Very high	−1.784	0.074
Grand mean	4.07 (0.79)	80.53	Very High	4.17 (0.83)	83.73	Very high	−3.912	0.004 **
Awareness								
When it comes to food, I am unconcerned with eating healthy	3.19 (1.09)	62.80	Medium	3.60 (1.00)	71.7	High	−3.167	0.002 **
I am very concerned that the food I eat is nutritious	3.60 (1.27)	75.60	High	3.87 (1.14)	71.9	High	−2.785	0.005 **
I eat food that I enjoy regardless of whether it is healthy or not	2.49 (1.09)	52.00	Low	2.27 (1.24)	51.8	Low	−2.967	0.003 **
The meals I eat must be low in fat	3.40 (1.13)	68.10	Medium	3.22 (1.18)	63.8	Medium	−2.424	0.015 *
I always eat a well-balanced and healthy diet	2.65 (1.23)	54.90	Low	2.44 (0.95)	50.7	Low	−1.910	0.056
My meal must be rich in vitamins and minerals	3.32 (1.29)	66.80	Medium	3.98 (0.94)	74.7	High	−0.869	0.385
I do not care whether the snacks that I eat are nutritious or not	2.52 (1.13)	54.70	Low	2.28 (1.33)	54.1	Low	−2.249	0.025 *
I do not avoid foods that raise my cholesterol	3.32 (1.09)	64.80	Medium	3.49 (1.31)	69.1	Medium	−4.520	0.0001 **
I do not want to think about whether the food I am eating is beneficial for me or not	3.36 (1.39)	67.60	Medium	3.29 (1.37)	68.6	Medium	−3.337	0.001 **
I am willing to forego certain items in order to focus on a healthy diet	3.46 (1.37)	63.30	Medium	3.63 (1.05)	72.2	High	−1.962	0.050 *
It is critical to understand how to eat a healthy diet	3.41 (1.29)	69.40	Medium	3.69 (0.83)	72.5	High	−2.154	0.031 *
Other individuals, I feel, are more concerned with good eating than I am	2.20 (1.46)	51.40	Low	2.39 (1.24)	54.2	Low	−1.179	0.238
I believe that eating fruits and vegetables five times per day is essential	3.36 (1.25)	69.30	Medium	3.69 (0.90)	74.9	High	−3.714	0.0001 **
I am concerned about not ingesting too much food	3.21 (1.39)	68.20	Medium	3.50 (1.23)	66.7	Medium	−0.465	0.642
I am concerned about eating a well-balanced diet	4.17 (0.74)	83.30	Very High	3.47 (1.28)	66.7	Medium	−0.692	0.489
I am quite concerned about eating my meals regularly	3.59 (1.19)	69.10	Medium	3.50 (1.13)	64.5	Medium	−0.042	0.967
I am concerned about not drinking excessive amounts of sugar	3.68 (0.87)	74.70	High	4.07 (0.76)	82.4	Very high	−1.019	0.308
Grand mean	3.23 (1.34)	65.65	Medium	3.32 (1.26)	66.50	Medium	−2.322	0.013 *

* *p* ≤ 0.05 and ** *p* ≤ 0.01.

**Table 4:** Spearman correlation coefficients (*r*) and simple linear regression analysis (*β*, *r*^2^) between demographic characteristics and food label knowledge for males (*n* = 360).

Independent Variable/Dependent Variable	Marital Status		Age		Educational Level		Monthly Income		Living Status	
	*r*	(*β*, *r*^2^)	*r*	(*β*, *r*^2^)	*r*	(*β*, *r*^2^)	*r*	(*β*, *r*^2^)	*r*	(*β*, *r*^2^)
Calories are a unit of measurement for the amount of energy in food;	0.014	(0.005, 0.001)	0.040	(0.038, 0.041)	0.007	(0.024, 0.005)	0.006	(0.041, 0.050)	−0.150 **	(−0.032 **, 0.005)
Keeping track of the number of calories taken in food can help you achieve your optimum weight;	0.041	(0.012, 0.033)	−0.007	(−0.040, 0.041)	0.006	(0.020, 0.063)	−0.045	(−0.040, 0.012)	−0.021 *	(−0.040 *, 0.002)
Calories are found in vitamins and minerals;	−0.026	(−0.006, 0.015)	0.036	(0.019, 0.030)	0.046	(0.016, 0.005)	0.033	(0.011, 0.003)	−0.038 *	(−0.016 *, 0.026)
Calories are found in carbohydrates, proteins, and lipids;	−0.009	(−0.042, 0.024)	−0.071	(−0.055, 0.015)	−0.080	(−0.094, 0.031)	−0.069	(−0.111, 0.069)	0.009	(0.039, 0.034)
In terms of calories provided, fat and carbohydrates vary;	−0.026	(−0.003, 0.025)	0.067	(0.001, 0.001)	0.060	(0.019, 0.017)	0.025	(0.038, 0.007)	0.041	(0.022, 0.031)
The daily calorie requirements of an individual are affected by various factors, including weight, height, age, gender, and physical activity;	−0.156 **	(−0.067 **, 0.014)	0.084	(0.014, 0.017)	0.148 **	(0.110 **, 0.025)	0.073	(0.059, 0.003)	0.083	(0.111, 0.09)
The Saudi Food and Drug Authority ordered restaurants and coffee shops to include calorie information on their menus;	0.076	(0.007, 0.021)	−0.103 *	(−0.071 *, 0.045)	0.077	(0.016, 0.027)	−0.064	(−0.080, 0.007)	−0.035 *	(−0.010 *, 0.009)
When ordering from a restaurant, it is a good idea to check the calorie information on the menu;	0.095	(0.016, 0.009)	−0.047	(−0.127, 0.052)	0.009	(0.059, 0.019)	−0.040	(−0.029, 0.038)	−0.020 *	(−0.035 *, 0.041)
Calorie labels on restaurant menus are simple to understand;	−0.001	(−0.012, 0.016)	0.032	(0.022, 0.030)	0.041	(0.022, 0.008)	0.032	(−0.005, 0.008)	−0.020 *	(0.079 *, 0.079)
Adults require approximately 2000 calories per day on average;	0.048	(0.028, 0.025)	0.008	(0.110, 0.056)	0.076	(0.018, 0.003)	−0.029	(−0.040, 0.054)	−0.029 *	(−0.028 *, 0.012)
Individuals’ daily calorie requirements vary from one another;	0.087	(0.001, 0.023	0.092	(0.090, 0.053)	0.082	(0.026, 0.017)	−0.032	(−0.043, 0.045)	−0.050 *	(−0.066 *, 0.046)
Knowing the number of calories consumed aids in adding balance and utilized energy in the body	0.054	(0.008, 0.015)	0.012	(0.032, 0.013)	0.057	(0.034, 0.026)	−0.031	(−0.033, 0.039)	0.058 *	(0.025 *, 0.0021)

* *p* ≤ 0.05 and ** *p* ≤ 0.01.

**Table 5:** Spearman correlation coefficients (*r*) and simple linear regression analysis (*β*, *r*^2^) between demographic characteristics and food label knowledge for females (*n* = 442).

Independent/Dependent Variables	Marital Status		Age		Educational Level		Monthly Income		Living Status	
	*r*	(*β*, *r*^2^)	*r*	(*β*, *r*^2^)	*r*	(*β*, r^2^)	*r*	(*β*, *r*^2^)	*r*	(*β*, *r*^2^)
Calories are a unit of measurement for the amount of energy in food;	−0.017 *	(−0.018 *, 0.041)	−0.002	(−0.014, 0.018)	−0.116 *	(−0.011 *, 0.022)	−0.431 **	(−0.317 **, 0.269)	0.001	(−0.018, 0.002)
Keeping track of the number of calories taken in food can help you achieve your optimum weight;	−0.009	(−0.008, 0.002)	−0.014	(−0.011, 0.013)	−0.046	(−0.016, 0.027)	−0.039	(−0.036, 0.036)	−0.008	(−0.008, 0.001)
Calories are found in vitamins and minerals;	0.079	(0.022, 0.067)	0.070	(0.036, 0.064)	−0.149 **	(−0.018 **, 0.026)	0.053	(0.031, 0.045)	−0.050	(−0.022, 0.067)
Calories are found in carbohydrates, proteins, and lipids;	0.144 **	(0.079 **, 0.020)	0.042	(0.052, 0.055)	0.101 *	(0.036, 0.056)	0.037	(0.058, 0.050)	0.062 **	(0.079 **, 0.143)
In terms of calories provided, fat and carbohydrates vary;	−0.095 *	(−0.040 *, 00.011)	−0.049	(−0.036, 0.054)	−0.218	(−0.022, 0.002)	−0.049	(−0.048, 0.003)	−0.033	(−0.010, 0.037)
The daily calorie requirements of an individual are affected by various factors, including weight, height, age, gender, and physical activity;	0.117 *	(0.065 *, 0.031)	0.080	(0.059, 0.055)	0.059	(0.021, 0.028)	0.132 **	(0.162 **, 0.125)	0.085 *	(0.039 *, 0.008)
The Saudi Food and Drug Authority ordered restaurants and coffee shops to include calorie information on their menus;	0.117 **	(0.057 **, 0.013)	0.125 **	(0.129 **, 0.022)	0.043 **	(0.077 **, 0.017)	0.039 *	(0.066 *, 0.062)	−0.003	(−0.003, 0.002)
When ordering from a restaurant, it is a good idea to check the calorie information on the menu;	−0.031	(−0.022, 0.049)	−0.056	(−0.057, 0.005)	−0.032	(−0.038, 0.072)	−0.053	(−0.037, 0.039)	0.026	(0.017, 0.003)
Calorie labels on restaurant menus are simple to understand;	0.080	(0.042, 0.099)	0.095 *	(0.103 *, 0.019)	0.037	(0.028, 0.057)	0.137 **	(0.142 **, 0.025)	0.005	(0.004, 0.001)
Adults require approximately 2000 calories per day on average;	0.111 *	(0.041 *, 0.011)	−0.050 *	(−0.035 *, 0.052)	0.062	(0.057, 0.011)	0.028	(0.040, 0.011)	−0.021	(−0.007, 0.001)
Individuals’ daily calorie requirements vary from one another;	0.130 **	(0.094 **, 0.023)	0.074	(0.091, 0.084)	0.080	(0.069 *, 0.095)	0.091 *	(0.148 *, 0.113)	0.028	(0.021, 0.002)
Knowing the number of calories consumed aids in adding balance and utilized energy in the body	0.074	(0.041, 0.080)	0.020	(0.011, 0.012)	0.044	(0.029, 0.010)	0.053	(0.062, 0.058)	0.010	(0.010, 0.001)

* *p* ≤ 0.05 and ** *p* ≤ 0.01.

**Table 6:** Spearman correlation coefficients (*r*) and simple linear regression analysis (*β*, *r*^2^) between demographic characteristics and food policy awareness after calorie labeling for males (*n* = 360).

Independent/Dependent Variable	Marital Status		Age		Educational Level		Monthly Income		Living Status	
	*r*	(*β*, *r*^2^)	*r*	(*β*, *r*^2^)	*r*	(*β*, *r*^2^)	*r*	(*β*, *r*^2^)	*r*	(*β*, *r*^2^)
When it comes to food, I am unconcerned with eating healthy;	0.123 *	(0.025 *, 0.014)	0.181 **	(0.081 **, 0.178)	0.164 **	(0.042 **, 0.026)	0.129 *	(0.077 *, 0.018)	0.149 **	(0.039 **, 0.146)
I am very concerned that the food I eat is nutritious;	−0.181 **	(−0.043 **, 0.031)	−0.193 **	(−0.102 **, 0.038)	−0.172 **	(−0.049 **, 0.027)	−0.121 *	(−0.086 *, 0.107)	−0.044	(−0.011, 0.036)
I eat food that I enjoy regardless of whether it is healthy or not;	0.073	(0.014, 0.064)	0.187 **	(0.078 **, 0.028)	0.168 **	(0.044 **, 0.027)	0.201 **	(0.115 **, 0.093)	0.025	(0.009, 0.001)
The meals I eat must be low in fat;	−0.141 **	(−0.033 **, 0.019)	−0.110 *	(−0.053 *, 0.105)	−0.123 *	(−0.033 *, 0.014)	−0.019	(−0.005, 0.008)	0.033	(0.015, 00.002)
I always eat a well-balanced and healthful diet;	−0.171 **	(−0.036 **, 0.025)	−0.228 **	(−0.098 **, 0.199)	−0.228 **	(−0.062 **, 0.050)	−0.172 **	(−0.10 8 **, 0.031)	0.081	(0.025, 0.086)
My meal must be rich in vitamins and minerals;	−0.181 **	(−0.044 **, 0.030)	−0.163 **	(−0.087 **, 0.026)	−0.152 **	(−0.046 **, 0.023)	−0.072	(−0.065, 0.097)	0.029	(0.010, 0.031)
I do not care whether the snacks are nutritious or not;	0.053	(0.010, 0.046)	0.071	(0.029, 0.004)	0.111 *	(0.028 *, 0.011)	0.102	(0.057, 0.097)	0.010	(0.003, 0.009)
I do not avoid foods that raise my cholesterol;	0.138 **	(0.030 **, 0.018)	0.199 **	(0.090 **, 0.037)	0.212 **	(0.055 **, 0.043)	0.132 *	(0.076 *, 0.130)	0.020	(0.007, 0.001)
I do not want to think about whether the food I am eating is beneficial for me or not;	−0.008	(−0.001, 0.005)	0.092	(0.037, 0.082)	0.086	(0.021, 0.084)	0.080	(0.047, 0.084)	0.082	(0.021, 0.079)
I am willing to forego certain items in order to focus on a healthy diet;	−0.122 **	(−0.032 **, 0.014)	−0.141 **	(−0.082 **, 0.145)	−0.102	(−0.029, 0.091)	−0.132 **	(−0.086 **, 0.015)	0.047 *	(0.020 *, 0.004)
It is critical to understand how to eat a healthy diet;	−0.060	(−0.021, 0.070)	−0.064	(−0.058, 0.089)	−0.075	(−0.033, 0.089)	0.080	(−0.044, 0.054)	0.001	(0.002, 0.004)
Other individuals, I feel, are more concerned with good eating than I am;	−0.038	(−0.009, 0.038)	0.001	(−0.003, 0.006)	0.007	(0.003, 0.010)	0.025	(0.011, 0.017)	0.105 *	(0.032 *, 0.110)
I believe that eating fruits and vegetables five times per day is essential;	−0.034	(−0.008, 0.001)	−0.038	(−0.013, 0.021)	−0.028	(−0.010, 0.028)	−0.025	(−0.046, 0.058)	−0.049	(−0.016, 0.044)
I am concerned about not ingesting too much food;	−0.043	(−0.012, 0.003)	−0.067	(−0.036, 0.074)	−0.050	(−0.014, 0.051)	0.021	(0.002, 0.004)	−0.069	(−0.017, 0.061)
I am concerned about eating a well-balanced diet;	−0.048	(−0.012, 0.002)	−0.047	(−0.019, 0.001)	−0.062	(−0.019, 0.062)	−0.069	(−0.048, 0.072)	−0.048	(−0.012, 0.001)
I am quite concerned about eating my meals regularly;	−0.105 *	(−0.024 *, 0.100)	−0.109 *	(−0.059 *, 0.014)	−0.146 **	(−0.041 **, 0.021)	−0.026	(−0.017, 0.027)	0.030	(0.013, 0.043)
I am concerned about not drinking excessive amounts of sugar	−0.045	(−0.012, 0.050)	−0.086	(−0.028, 0.003)	−0.079	(−0.025, 0.086)	−0.028	(−0.033, 0.051)	−0.017	(0.016, 0.001)

* *p* ≤ 0.05 and ** *p* ≤ 0.01.

**Table 7:** Spearman correlation coefficients (*r*) and simple linear regression analysis (*β*, *r*^2^) between demographic characteristics and food policy awareness after calorie labeling for females (*n* = 442).

Independent/Dependent Variable	Marital Status		Age		Educational Level		Monthly Income		Living Status	
	*r*	(*β*, *r*^2^)	*r*	(*β*, *r*^2^)	*r*	(*β*, *r*^2^)	*r*	(*β*, *r*^2^)	*r*	(*β*, *r*^2^)
When it comes to food, I am unconcerned with eating healthy	0.146 **	(0.047 **, 0.017)	0.119 *	(0.080 **, 0.016)	0.121 *	(0.054 *, 0.016)	−0.012	(−0.007, 0.009)	−0.081	(−0.020, 0.007)
I am very concerned that the food I eat is nutritious	−0.171 **	(−0.071 **, 0.027)	−0.215 **	(−0.163 **, 0.047)	−0.178 **	(−0.085 **, 0.028)	−0.085	(−0.085, 0.094)	0.062	(0.018, 0.060)
I eat food that I enjoy regardless of whether it is healthy or not;	0.096 *	(0.034 *, 0.008)	0.127 **	(0.087 **, 0.017)	0.153 **	(0.066 **, 0.022)	−0.045 *	(−0.032 *, 0.039)	−0.074	(−0.022, 0.007)
The meals I eat must be low in fat	−0.086	(−0.034, 0.083)	−0.078	(−0.058, 0.007)	−0.036	(−0.020, 0.002)	−0.079	(−0.059, 0.005)	0.122 *	(0.032 *, 0.013)
I always eat a well-balanced and healthful diet	−0.054	(−0.020, 0.003)	−0.158 **	(−0.116 **, 0.027)	−0.083	(−0.039, 0.081)	−0.051	(−0.049, 0.057)	0.019	(0.005, 0.019)
My meal must be rich in vitamins and minerals	−0.093 *	(−0.041 *, 0.096)	−0.107 *	(−0.085 *, 0.116)	−0.091 *	(−0.049 *, 0.097)	0.028	(0.020, 0.001)	0.047	(0.015, 0.002)
I do not care whether the snacks that I eat are nutritious or not	0.061	(0.020, 0.057)	0.102 *	(0.064 *, 0.010)	0.094 *	(0.039 *, 0.093)	−0.005	(−0.004, 0.006)	−0.096 *	(−0.023 *, 0.093)
I do not avoid foods that raise my cholesterol	0.046	(0.017, 0.002)	0.053	(0.030, 0.002)	0.096 *	(0.042 *, 0.101)	−0.017	(−0.011, 0.015)	−0.143 **	(−0.037 **, 0.023)
I do not want to think about whether the food I am eating is beneficial for me or not	0.026	(0.008, 0.001)	0.044	(0.025, 0.002)	0.071	(0.033, 0.007)	−0.087	(−0.047, 0.066)	−0.099 *	(−0.023 *, 0.098)
I am willing to forego certain items to focus on a healthy diet	−0.062	(−0.022, 0.003)	−0.053	(−0.033, 0.046)	−0.002	(−0.002, 0.005)	−0.010	(−0.007, 0.008)	0.032	(0.012, 0.041)
It is critical to understand how to eat a healthy diet	0.031	(0.012, 0.001)	−0.036	(−0.025, 0.027)	−0.026	(−0.013, 0.022)	−0.032	(−0.022, 0.020)	0.057	(0.040, 0.110)
Other individuals, I feel, are more concerned with good eating than I am	0.040	(0.013, 0.001)	0.101 *	(0.061 *, 0.098)	0.043	(0.016, 0.037)	−0.032	(−0.024, 0.032)	−0.031	(−0.008, 0.001)
I believe that eating fruits and vegetables five times per day is essential	−0.008	(−0.013, 0.001)	−0.031	(−0.051, 0.003)	−0.055	(−0.045, 0.072)	−0.082	(−0.092, 0.081)	0.030	(0.013, 0.001)
I am concerned about not ingesting enough food	−0.043	(−0.021, 0.003)	−0.042	(−0.030, 0.002)	−0.045	(−0.023, 0.003)	−0.065	(−0.069, 0.008)	−0.003	(−0.001, 0.005)
I am concerned about eating a well-balanced diet	−0.072	(−0.031, 0.005)	−0.137 **	(−0.094 **, 0.015)	−0.105 *	(−0.054 *, 0.104)	−0.089 *	(−0.085 *, 0.091)	0.093	(0.030, 0.098)
I am quite concerned about eating my meals regularly	−0.117 *	(−0.045 *, 0.014)	−0.109 *	(−0.060 *, 0.092)	−0.136 **	(−0.059 **, 0..017)	−0.103 *	(−0.081 *, 0.101)	0.089	(0.027 *, 0, 0.102)
I am concerned about not drinking excessive amounts of sugar	−0.049	(−0.014, 0.002)	−0.102 *	(−0.044 *, 0.073)	−0.128 **	(−0.048 *, 0.014)	−0.024	(−0.023, 0.001)	−0.061	(−0.012, 0.003)

* *p* ≤ 0.05 and ** *p* ≤ 0.01.

## Data Availability

The datasets used and analyzed in the current study are available from the corresponding author upon reasonable request.
